# Digital Micro Interventions for Behavioral and Mental Health Gains: Core Components and Conceptualization of Digital Micro Intervention Care

**DOI:** 10.2196/20631

**Published:** 2020-10-29

**Authors:** Amit Baumel, Theresa Fleming, Stephen M Schueller

**Affiliations:** 1 University of Haifa Haifa Israel; 2 Victoria University of Wellington Wellington New Zealand; 3 University of California, Irvine Irvine, CA United States

**Keywords:** micro intervention, mental health, mhealth, eHealth, engagement, intervention, adherence, behavior change, behavioral health

## Abstract

Although many people access publicly available digital behavioral and mental health interventions, most do not invest as much effort in these interventions as hoped or intended by intervention developers, and ongoing engagement is often low. Thus, the impact of such interventions is minimized by a misalignment between intervention design and user behavior. Digital micro interventions are highly focused interventions delivered in the context of a person’s daily life with little burden on the individual. We propose that these interventions have the potential to disruptively expand the reach of beneficial therapeutics by lowering the bar for entry to an intervention and the effort needed for purposeful engagement. This paper provides a conceptualization of digital micro interventions, their component parts, and principles guiding their use as building blocks of a larger therapeutic process (ie, digital micro intervention care). The model represented provides a structure that could improve the design, delivery, and research on digital micro interventions and ultimately improve behavioral and mental health care and care delivery.

## Introduction

Mental illnesses and substance use disorders constitute a major public health challenge, with large personal social and economic costs [1-3]. Evidence-based interventions exist; however, most people with mental or behavioral health issues receive no treatment, and for those who do receive care, the average duration of untreated illness is excessive [3-6]. This treatment gap is linked to psychological and social barriers, such as feelings of embarrassment, stigma, and shame [7], as well as structural barriers, such as pricing and inconvenient time or location of services [2,3,8-10].

Technology-enabled intervention delivery models have grown on the promise to increase access to care. Behavioral and mental health apps can often be accessed without requiring others to be aware of personal behavioral or mental health issues, without physically attending appointments, and with considerably lower cost than that of face-to-face services. Self-help apps and programs, in particular, can often be accessed with no referral and be available 24/7. The reduction of barriers associated with traditional service access is important for improving access to care; however, it also brings some challenges. Those who make it to traditional services have often invested considerable effort to seek and engage with services and hence might often have high levels of motivation. For example, one study found that most outpatients surveyed prior to entering psychotherapy tended to believe they will be highly involved, invest large efforts, and make large changes in their life [11].

Models of care delivery that eliminate key barriers might reach an audience with very different characteristics, interests, and motivation than those reached by traditional services. Nowhere is this more evident than in the real-world usage of self-guided digital behavioral and mental health interventions situated outside of traditional treatment settings. A recent study examined the real-world use of popular mental health apps and reported that after 15 days of use, the median percentage of daily active users (open rate) was 4.0%, with a median app retention rate of 3.9% [12]. These findings are congruent with researchers’ own reports of poor real-world program use [13]. Subsequently, a recent systematic comparison of published reports and real-world usage of the same programs reported that users who participated in trials had four times higher eHealth program usage in comparison with real-world users of the same programs [14]. These findings imply that a high portion of those interested in digital behavioral and mental health interventions in the real world are not investing as much effort in these interventions as intended by intervention developers.

Many efforts have attempted to overcome this challenge, including efforts to address content packaging and personalization of treatment [15-17] and efforts to investigate the impact of intervention design features on engagement [18-21]. Another way of thinking, however, would be to accept that people might enter digital behavioral or mental health interventions with reduced levels of interest and motivation and to design interventions appropriate for such people. One potential modification to interventions would be to make interventions shorter and much more focused. These shorter and more focused interventions have been called “micro interventions” [22,23], which are highly focused interventions delivered in the context of a person’s daily life in order to help them reach desired proximal targets. These small units of beneficial therapeutics can be delivered with little burden on the individual. Therefore, micro interventions have the potential to not only lower the bar for entry to an intervention but also the commitment and effort needed for purposeful engagement.

People commonly seek and receive micro interventions all the time. Receiving an answer to a question posted in a Facebook community about parenting could be viewed as a micro intervention. Playing an uplifting song may assist individuals with emotion regulation and could also be viewed as a micro intervention. Micro interventions are not unique to digital interventions, however, new technological affordances, such as the widespread penetration of smartphones [24,25] and the global increase in internet access [26,27], present opportunities to easily offer digital micro interventions in people’s natural context. These digital micro interventions could substantially increase access to effective behavioral and mental health care by lowering the amount of effort required to reach beneficial gains.

Despite the accessibility of these affording technologies and the frequency of digital micro interventions being deployed daily, a conceptualization of digital micro interventions and how to effectively integrate them within larger therapeutic processes has not been offered. In this paper, we aim to address this gap. We begin by defining a digital micro intervention and its component parts. We then describe principles guiding the use of digital micro interventions as building blocks of a larger therapeutic process (ie, digital micro intervention care). We close by clarifying the differences between digital micro intervention care and a stepped-care approach. Table 1 provides an explanatory overview of key terms described in the body of this work. Through this paper, we hope to help create a language and conceptualization that enables scholars to clarify the importance of specific digital micro interventions within larger processes and to support the creation of digital micro intervention care and the publication of studies around them.

**Table 1:** Key terms described within the paper.

Term		Definition	Example
**Digital micro intervention**		An intervention intended to achieve a highly focused objective using in-the-moment elements. These elements are not necessarily linked directly to the achievement of a larger clinical aim.	Guiding parents in several small steps to help them increase positive attention toward desired behaviors of their child.
	Events	The elemental (smallest) components of digital micro interventions. Each event is an in-the-moment attempt for change or impact toward the overall target of the intervention.	An app-based gratitude exercise encouraging parents to focus on their child’s positive attributes.
	Decision rules	Guiding which events are deployed and when.	Including a short educational event before deploying a series of repeating in-the-moment gratitude exercises.
	Proximal assessments	Assessing the impact of the event.	Short educational event might correspond to the proximal outcomes of increasing knowledge and motivation.
	Overall micro intervention outcome	The target of the micro intervention which is not likely the same as the overall clinical goals.	Reaching parental sustainable positive attention toward the child (and not reduction in symptoms of the child’s behavior problems).
**Digital micro intervention care**		Using digital micro interventions as building blocks of a larger therapeutic process aimed toward a target outcome.	Providing different digital micro interventions within a long process of helping parents develop emotional and social competences in their child.
	Micro interventions	The building blocks of this model of care. See definition of micro intervention above.	See example for a digital micro intervention in the first row.
	Conceptual model of the therapeutic process	Defines how digital micro interventions can address steps within the therapeutic process; utilized to identify the relevant digital micro interventions and the context in which they should be used.	Providing a rationale as to how emotional/social competences develop and what issues should be prioritized based on established concepts.
	Therapeutic narrative as a linking bridge between interventions	A narrative presented to the user serving as a linking bridge enabling to move from one digital micro intervention to another in a way that consolidates the experience of the different interventions.	An automated prewritten text sent to parents that acknowledges their success in completing two past micro interventions; explains the rational for the current intervention and in what ways this new intervention is meaningful.
	Hub	Centralizes and links between the separate micro interventions; delivers micro interventions as well as the therapeutic narrative based on the conceptual model and an evaluation of user context/needs.	See the “One Hub to Enable Proper Digital Micro Intervention Care” section.

## Defining a Digital Micro Intervention and Its Component Parts

### Overview

Micro interventions differ from standard (ie, more extended) and brief interventions in their breadth, goals, and time frame. Standard and brief interventions are designed to achieve a broad clinical aim (eg, overcome depression and gain desired weight loss) by providing consumers with a complete package of elements [28-30]. Micro interventions, by comparison, are narrower in scope and intended to achieve a highly focused objective [22,23], using in-the-moment elements to promote emotional, cognitive, or behavioral change. These in-the-moment changes are not necessarily linked directly to the achievement of a larger clinical aim, although they may have implications or contributions to such an aim. For example, while a parent training program may aim to target reducing a child’s disruptive behaviors as the overall goal (standard intervention), a digital micro intervention will target a much more focused aim such as guiding the parents in several small steps to help them increase their positive attention toward desired behaviors of their child. Because these interventions are highly focused, in most cases, their time frame will be shorter than other interventions.

We propose that digital micro interventions are based on the following three core components: events (the individual outreaches to the target of the digital micro intervention), decision rules (guiding which events should be deployed and when), and assessments (determining the impact of the event and the intervention). In what follows, we will describe these components in detail.

### Events

Events are the elemental components of digital micro interventions. Each event is an in-the-moment attempt for change or impact toward the overall target of the intervention. A digital micro intervention may be based on one event [31,32] or multiple events [23,33]. Table 2 displays common goals of events along with associated definitions and examples. Events can be informational, such as providing didactic material in the moment; interventional, such as attempting to spur some form of change or action in the moment like calm breathing; or supportive, such as providing some encouragement, understanding, or social support. The critical aspect is that the end point for benefit tends to be near term. For example, an informational event should be something that can be taught quickly.

**Table 2:** Common goals of digital micro intervention events, descriptions, and related examples.

Goals/targets	Description	Example
		
Educational	Didactic material or psychoeducation intended to teach someone something in the moment.	A video teaching a parent how to discuss the importance of a certain behavior with a child. A timely text message motivating a person to conduct a physical activity.
Feedback	Providing information in the moment to reflect an individual’s current state in a beneficial way.	Feedback about how one’s current activity compares to activity at another time, such as you walked 15,000 steps today, which is 5000 more than average.
Change of perspective (eg, reframing)	Tunnel individual’s focus or provoke thought processes intended to engage a person with a different perspective.	An app-based gratitude exercise encouraging an individual to focus on one’s positive attributes, to increase in-the-moment satisfaction [22].
Trigger desired action	Reminder or incentive to get people to engage in a concrete action in the moment.	A notification from a wearable device suggesting that an individual stand [34].
Skill acquisition	Providing support in skill acquisition.	In-the-moment guidance on breathing exercise.
Load reduction of therapeutic-related activity	Enabling the manifestation of a beneficial activity through simplification.	In-the-moment report on calorie intake using a smartphone camera to capture a photo of food.
Symptom relief	Providing relief of negative symptoms.	An app helping a person to identify and perform competing activities to reduce in-the-moment desire for binge drinking.
Social/emotional support	Providing or indicating support from an actual or perceived other.	A text message indicating support or understanding.

### Decision Rules

**Figure 1:**
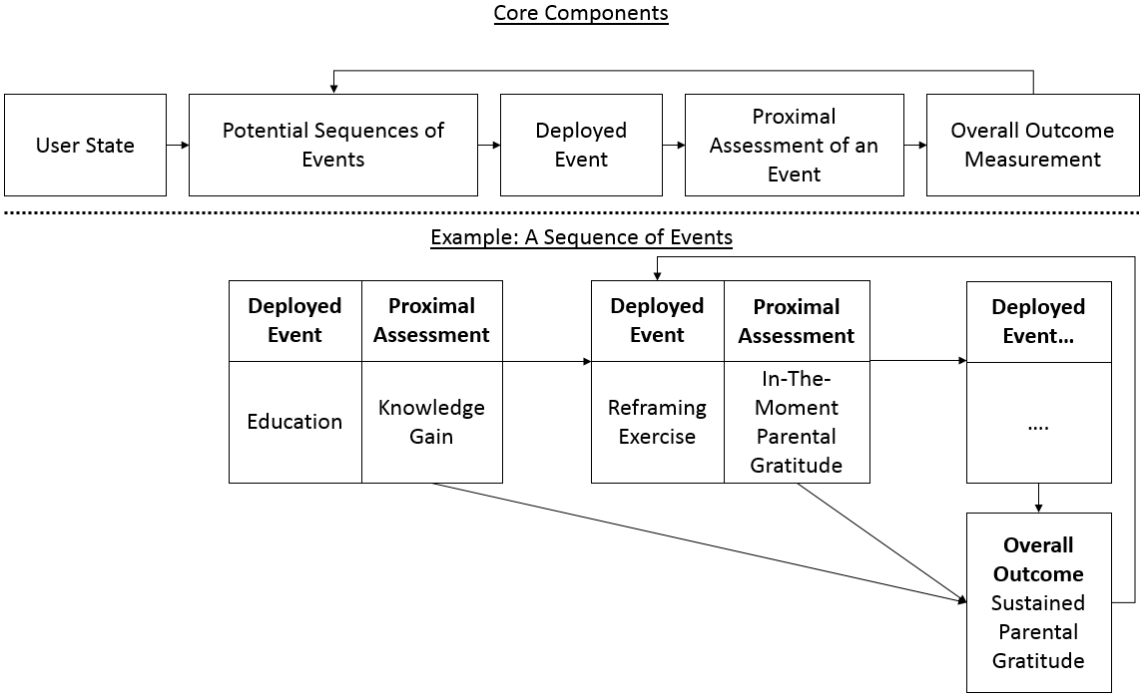
Illustration of a sequence of events based on decision rules.

Decision rules refer to specifications regarding which events should be deployed and when. They sequence and combine events in meaningful ways to create the micro intervention. When decision rules deploy events according to time-based, user-based, or environment-based information, the digital micro interventions can also be referred to as either ecological momentary interventions [35,36] or just-in-time adaptive interventions [37]. The sequence of events that follow from decision rules is closely tied to the intervention developer’s understanding of the mechanistic underpinnings of how the micro intervention is perceived to operate )see Figure 1 for a suggested illustration(. For example, if an intervention developer designs an intervention with the goal of achieving sustained parental gratitude, including a short educational event before a series of repeating in-the-moment exercises, the developer assumes that knowledge or understanding of the therapeutic rational is necessary prior to the interactive exercises.

This notion of a digital micro intervention as an effective sequence of events has conceptual overlap with the notion of evidence-based kernels of Embry and Bilgan [38]. In their definition, kernels are influential elements, which constitute “fundamental units of behavior influence in the sense that deleting any component of a kernel would render it inert.” Similarly, the right combination of events is responsible for producing the digital micro intervention’s outcome. As such, both the proximal assessments of events and the general outcome of the micro intervention can feed into decision rules to guide future events. Decision rules may also incorporate contextual elements, including a user’s state, environmental variables, or previously identified user characteristics, to allow dynamic tailoring of the micro intervention’s content [39,40]. For example, if parental gratitude is not sustained in individuals who do not achieve knowledge gain before a successful exercise event routine, it might mean that for individuals who fail in the first event (knowledge gain), there is no need to continue with the planned sequence. Alternatively, it might be that some individuals do not require a gain in knowledge prior to effective exercise events, and therefore, identifying them earlier based on their prior characteristics would enable to offer alternative paths within the same micro intervention. Indeed, contextual variables appear to be important predictors of the likelihood a person will respond to a prompt and engage with the intervention [41].

### Assessments

Assessing a digital micro intervention’s impact can be divided into assessing the overall outcome of the intervention and assessing the more proximal outcomes of deployed events. The overall outcome for digital micro interventions can be quite varied, although it is important to differentiate between the intervention outcome and the overall clinical goals, which are likely not the same. A digital micro intervention for individuals with serious mental illness, for example, might focus on medication adherence [42] in the hopes of reducing clinical symptoms. In this case, each event included within the intervention would focus on steps and proximal outcomes related to medication adherence. For example, supporting reasons for taking medication might correspond to the proximal outcome of increasing motivation, and providing tailored reminders for dosing periods or schedules might correspond to the proximal outcome of decreasing the cognitive effort needed to remember to initiate the desired activity. Assessing the overall benefit of this digital micro intervention, therefore, should focus not on how the intervention results in an overall clinical goal (eg, reduced symptoms of serious mental illness), but rather on how it impacts medication adherence. Importantly, if we examine the impact of events, we can explore how much each event contributes to the proximal outcome as well as the overall digital micro intervention goal.

Another aspect that relates to assessments is the user’s engagement with the micro intervention. In digital micro interventions, user engagement can be documented continuously based on program usage and experience of engagement (ie, measuring the quality of attention, involvement, and immersion during program usage) [43-46]. These data could be key in identifying not only those who properly engage with the intervention, but also those who disengage without getting all they need from it, in order to feed intervention decision rules to re-engage the users.

In this section, we have defined a digital micro intervention and presented its component parts. Subsequently, we address aspects that relate to the provision of digital micro intervention care. These aspects require us to take into consideration principles guiding the use of digital micro interventions as building blocks of a larger therapeutic process.

## Digital Micro Intervention Care: Digital Micro Interventions as Building Blocks of a Larger Therapeutic Process

### Overview

As described above, typically, individual micro interventions have relatively specific targets, rather than being full standalone treatments. Therefore, when developers think of a digital micro intervention within a larger therapeutic process, they have to determine what makes the intervention relevant or helpful, which additional digital micro interventions are expected or may be required over time, and how to integrate between the separate interventions within one therapeutic framework. In this section, we aim to address principles that support such a successful integration.

### A Conceptual Model to Guide Which Digital Micro Interventions are Required and in What Context

In order to identify the relevant digital micro interventions and the context in which they should be used, developers need a conceptual model of the therapeutic process and how digital micro interventions can address steps within this process. As an illustration, consider the example of providing different digital micro interventions within a long process of helping parents develop emotional and social competences in their child. In order to identify the required interventions, it is first necessary to build a rationale as to how these competences develop and are maintained, and what issues should be prioritized. Table 3 presents such an example. The table is based on social learning and behavioral theories [47,48], which build on concepts such as the parenting pyramid offered in the Incredible Years program [49], and parental monitoring and prevention of child behavior problems [47]. According to the model portrayed in the table, there are levels of parenting behaviors (setting prioritization between basic and more advanced behaviors) and benefits for the child that occur when parents exercise these behaviors. The desired parenting behaviors are then used to identify the relevant digital micro interventions for each level (see examples in Table 3).

**Table 3:** An example for a model defining the capabilities parents are required to present in order to prevent behavior problems and promote social and emotional competence.

Priority level	Goal	Parent behavior	Examples for relevant digital micro interventions	Child gains	
				
First level	Parental availability	Presence; attention; monitoring	Notifying the parents to put their smartphone away when doing activities with their child; triggering to leave the child a positive note on the kitchen table to increase presence during working days.	Attachment; self-esteem; cooperation	
First level	Positive parenting practices	Positive involvement; positive modelling; conversations; play	Teaching how to play with a 4-year-old child through scenario-based learning.	Attachment; self-esteem; cooperation	
Second level	Problem solving/prevention	Social coaching; proactive identification of relevant guidelines; consultations	Brief online video guidance on how to coach a 3-year-old child during play on using their mouth instead of their hands.	Social skills; meeting their potential; motivation; accountability	
Third level	Dealing with acute negative symptoms	Neglecting unhelpful routines and parenting styles; embracing beneficial practices	Connecting the parent with a peer through an online community in order to find the right consequence for a child’s misbehavior.	Back to normative developmental cycle; illness prevention	

The prioritization portrayed in the levels also presents the context in which each intervention is mostly relevant. If a digital micro intervention care developer builds on this specific conceptualization, it would not make sense from their view to train parents to set clear limits before they are, for example, present in their children’s life. As such, the incorporation of a model helps developers avoid designing and presenting people with a specific micro intervention that is not contextually relevant with the larger perspective in mind.

### Nurturing a Therapeutic Narrative to Link Between Independent Interventions

The literature suggests the importance of a clear therapeutic pathway and rationale to the user’s therapeutic process and that relatability factors embedded within a digital intervention support a working alliance [50-53]. A theme can serve as a linking bridge enabling to move from one digital micro intervention to another in a way that consolidates between the different interventions. It is also essential because the right narrative would make the intervention more contextually relevant to the specific process the consumer is going through. For example, a parent who learned and conducted a gratitude exercise with the family around the dinner table may benefit if it were connected to the theme of overcoming a child’s noncompliance, when this digital micro intervention is part of the general aim of treating a child’s disruptive behaviors. A different narrative may be in place for the same gratitude-invoking intervention, when the larger goal is different, for example, increasing parenting satisfaction among veterans with posttraumatic stress disorder [54,55]. Another simple example would be a digital micro intervention teaching individuals to change or reframe negative and unhelpful thoughts, which could be used for a broad range of clinical problems [56-58]. Naturally, the binding therapeutic narrative between different digital micro interventions would be different based on the targeted clinical problem.

**Figure 2:**
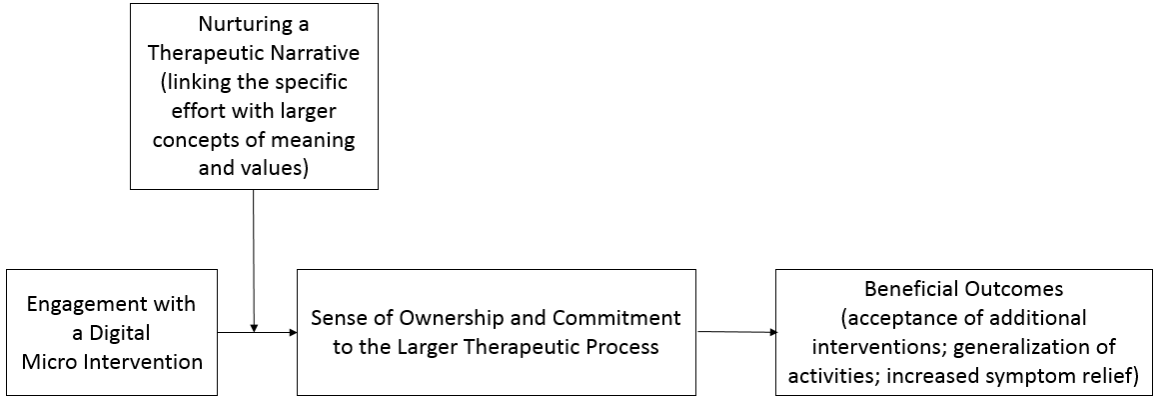
Nurturing a therapeutic narrative as a moderator to the connection between a digital micro intervention and sense of ownership and commitment to the therapeutic process.

Because the general process is the result of several digital micro interventions combined together, a therapeutic narrative may also have an important advantage as a moderator of the connection between each intervention and the sense of ownership and commitment toward the general process (see Figure 2 for a suggested illustration). This sense of ownership and commitment may, in turn, lead to beneficial outcomes such as an increase in motivation to invest in subsequent digital micro interventions in the same domain.

### One Hub to Enable Proper Digital Micro Intervention Care

The setting of multiple digital micro interventions within one therapeutic process requires a hub component in order to centralize and link between the separate interventions based on the principles discussed above. The hub is aimed at instilling a sense of accountability in the client [59] and at creating an integrated experience that relates to the therapeutic process, while taking an individual’s history into account. The hub meets these aims by (1) recognizing an individual’s state and context, (2) recommending interventions that are relevant, and (3) helping create and maintain the right narrative, meaning, and values that derive from each intervention and linking it to the larger therapeutic process.

The hub function could be performed by any entity that is set to meet these aims (a digital application, a human technology coach, a consumer who self-manages his or her state, or a psychotherapist). For example, in their study of the IntelliCare suite of apps aimed at treating depression and anxiety, Mohr and colleagues used a digital app as a hub that consolidated recommendations for the use of new apps [60]. Recommendations were based on a user’s current usage data of different apps within the IntelliCare suite to identify apps that the person will most likely use and find useful. Eventually, 95% of participants downloaded five or more IntelliCare apps as part of their therapeutic process, demonstrating both the technical feasibility and acceptability of this approach [60]. In another study, health technology coaches met occasionally with patients diagnosed with schizophrenia and suggested different digital tools based on individuals’ rehabilitation states and needs, and the impact of the tools they were using over time [61]. We could eventually see a new form of care, where a clinician (eg, a psychotherapist and psychiatrist) acts as a facilitator and meets with patients to suggest personalized digital micro interventions, including apps and online community support.

**Figure 3:**
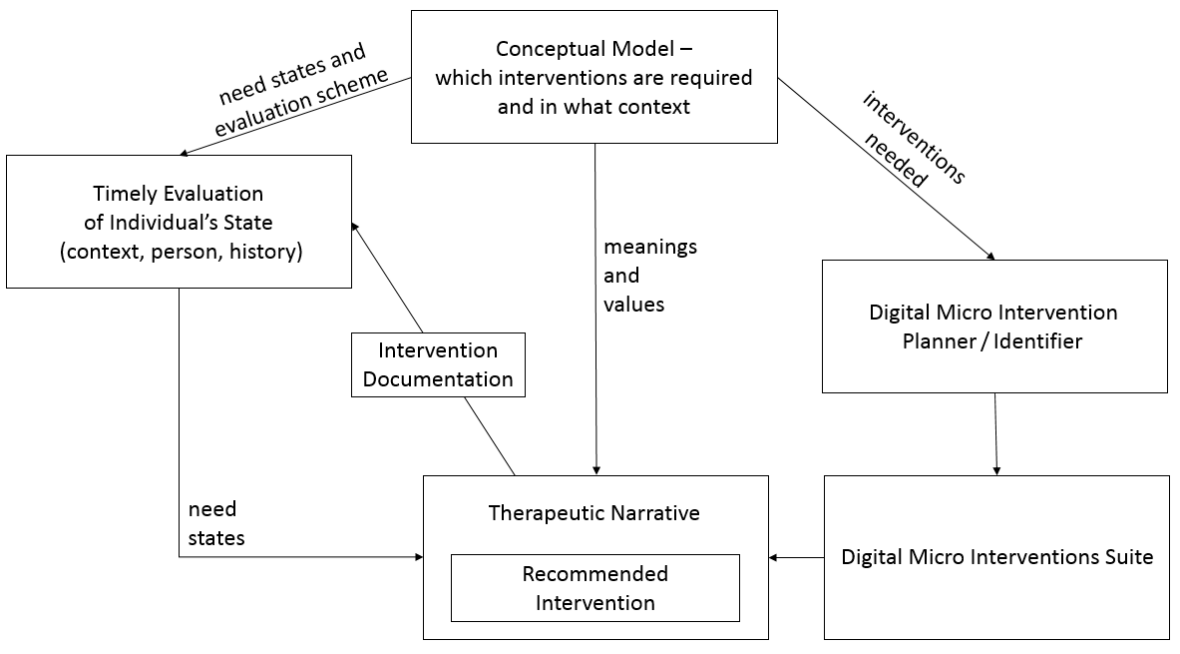
Visualization of the relationship between core components of digital micro intervention care.

Figure 3 presents a visualization of the relationship between the core components of digital micro intervention care and aims to represent how the facilitator (digital app, health technology coach, clinician, etc) integrates different interventions to curate one whole therapeutic process. The key components are as follows:

(1) *The conceptual model*: Defines which digital micro interventions are required, the relevant therapeutic narratives that link between different interventions, and the need states that also determine the evaluation scheme.

(2) *Digital micro intervention planner/identifier*: Encompasses the process of identifying the relevant interventions that are currently available and planning new interventions. The required interventions are defined based on the targeted need states determined within the conceptual model (see Table 3 for an example).

(3) *Digital micro intervention suite*: Includes all the potential interventions that are available. Once a need state is identified, the relevant intervention will be offered to the individual.

(4) *Timely evaluation of an individual’s state*: The evaluation scheme is based on data that relates to the individual (eg, symptoms and demographics), context (eg, changes in the environment that may change an individual’s needs), and details about past interventions and their outcomes. Need states can be alerted in two different ways. First, predefined rules can be made to trigger an evaluation based on a fixed time (eg, every 3 months) or context (eg, on a weekend when an individual is presumed to be more free or weekday when an individual is presumed to be working). Second, an individual can decide to report on his/her condition or the needs encountered, which will activate an evaluation.

(5) *Intervention phase*: The intervention phase is based on two different components. The basic component is the digital micro intervention itself. The second component represents the therapeutic narrative. The latter involves the following: (1) explaining the rationale for recommending the current micro intervention to the consumer, (2) clarifying how it relates to the general therapeutic process (if relevant based on the conceptual model), (3) integrating the current and past experiences (eg, success/failure in a previous intervention), and (4) relating to the future (eg, at the end of the intervention [what is going to happen next and why]) in relevant cases. Multimedia Appendix 1 presents an example of an automated therapeutic narrative that could be formed based on these subject matters.

(6) *Intervention documentation*: Summary data relating to engagement and outcomes are documented and used to feed the timely evaluation of an individual’s state. For example, if an individual disengages from an intervention without a clear change in symptoms and context, a different micro intervention can be offered.

### Ongoing Optimization

The success of the therapeutic process is limited by each of the units (micro interventions) within it and the ways they are linked and interact with each other. An iterative approach may enable the identification of those interventions that are missing or less effective, or a need to refine the conceptual model, for ongoing development of the intervention ecosystem. This paper’s focus is not on the methodological aspects of intervention optimization strategies; however, we refer the reader to the Multiphase Optimization Strategy as a good example for a methodological framework aimed at intervention optimization [62]. We find this framework to be relevant to digital micro intervention optimization because it is aimed at identifying the core sequence of elements that are efficacious, while providing a feasible method to account for potential intervening variables.

**Figure 4:**
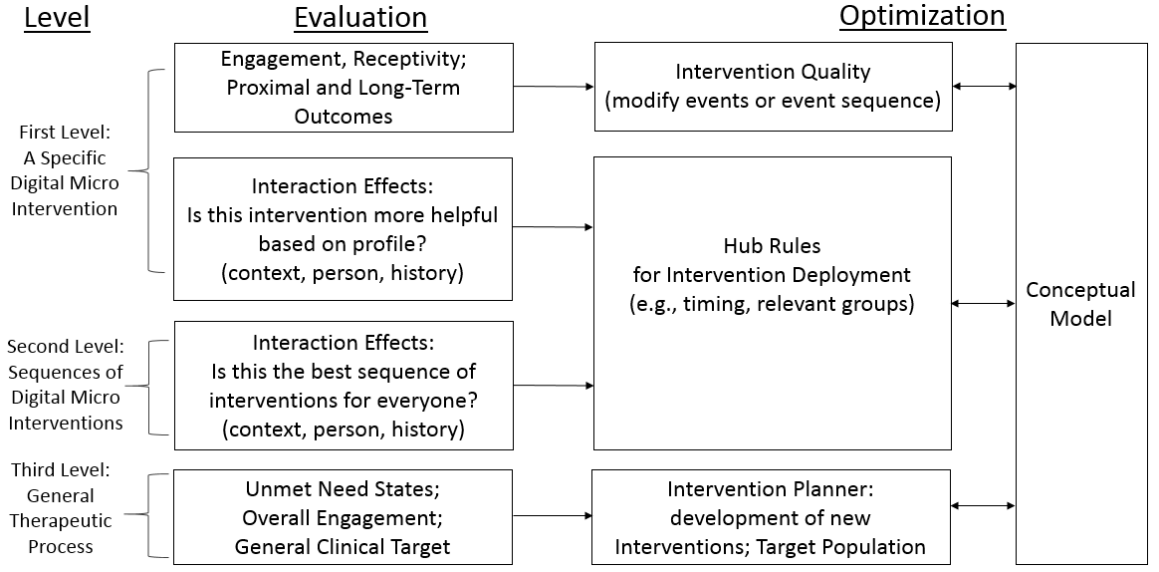
Levels of optimization.

Figure 4 presents the different areas of evaluation that could be expected in order to build on past knowledge to optimize the system of care based on several levels of examination. The first level of optimization focuses on a specific digital micro intervention. The receptivity and impact of the intervention is measured by user engagement, proximal events, and long-term outcomes. These results lead to optimization of the intervention’s quality, and may raise subsequent questions about the intervention’s impact that will require to add additional or alternative events or assessments. A subsequent evaluation involves the identification of specific populations and past experiences for whom/which the intervention was more or less successful (an interaction effect). The second level of optimization refers to the intervention sequence. Similar to the interaction effect examined in one digital micro intervention’s context, it involves identifying the best sequence of *interventions* that may differ based on the client’s context. The third and last level refers to the therapeutic process. It aims at understanding whether there are new need states that are not yet covered within the system, whether the clinical target is achieved, and in which cases. For example, if there are individuals who are properly engaged with the recommended sequence of micro interventions but do not reach the clinical target or individuals not engaged with any micro interventions that were offered. This will lead to optimizing the intervention planning and characterizing the user population for whom this system of care is relevant, and to the development of new interventions. The conceptual model is relevant in all levels of optimization because developers’ understanding of what works and for whom enables refinement of the model itself, which is then used to optimize the interventions and their sequence.

## Example: Digital Micro Intervention Care Versus a Stepped-Care Approach

In this final section of the paper, we compare the concept of digital micro intervention care to a stepped-care approach. Stepped care is a common conceptualization that combines a set of interventions within one therapeutic process [63-65]. A stepped care approach is defined by two main principles. First, the least intensive/restrictive treatment that is expected to provide the desired health gain is offered to the patient. Second, the model is adaptive in the sense that those individuals who do not reach a desired health gain in a predetermined time period will be assigned to a more intensive/restrictive treatment option. Therefore, a stepped-care approach is based on treatments designed to reach the desired health target, where these treatments differ in intensity. In contrast, a single digital micro intervention is not expected to support a full therapeutic target (eg, overcoming depression) but rather be a step along the way. This is reasonable given that when conceptualizing the use of multiple digital micro interventions, we do not assume that people are willing to invest as much effort as ideally needed to complete a whole target of therapeutic processes.

**Figure 5:**
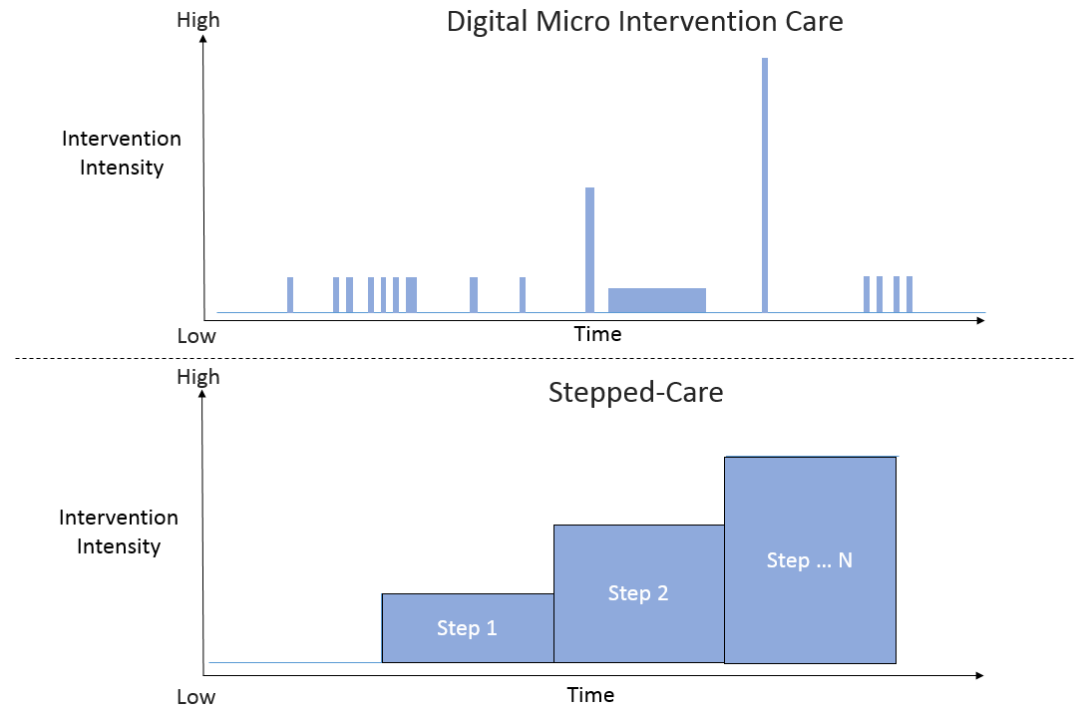
Illustrations of stepped care and digital micro intervention care models.

Figure 5 presents illustrations of the two frameworks, based on these underlying differences in delivery of care. In comparison to a stepped-care model, the following aspects are noted in digital micro intervention care: (1) Each intervention unit is highly focused and is generally short, reducing the effort needed for completion; (2) The setting of care does not prioritize intervention sequence based on treatment intensity, but rather based on a complex understanding of people’s needs and preferences (as described in the conceptual model section). Therefore, we do not see a graded intervention sequence in terms of intensity; (3) The therapeutic sequence does not focus on a given time window in which an intervention occurs continuously until completion, as would be expected in traditional models of proactive treatment delivery. This means that the therapeutic sequence may be very long and that individuals are sometimes engaged and sometimes not engaged with any intervention. In some cases, people do not receive any intervention most of the time. Maintaining ongoing contact with users may be required to allow triggering of the right intervention at the right time; (4) There might be a large number of different digital micro interventions to achieve the target of the treatment process, in comparison to stepped care models that are mostly based on few interventions (although each standard intervention includes multiple components).

## Conclusions

Digital micro interventions may provide new ways to deliver behavioral and mental health interventions while meeting people’s capacity to invest a small effort in beneficial therapeutics. The development of digital micro interventions, however, demands new thinking. We cannot merely take previous interventions based on other models and assume they will have the focused and immediate impacts required of micro interventions. As such, we have proposed a novel model of understanding digital micro interventions by identifying their component parts (events), how these parts work together (decision rules), and how digital micro interventions might be incorporated into a larger therapeutic process. This model is helpful to assist researchers in optimizing digital micro interventions, developing a conceptual understanding of each intervention’s role within a larger process, and leaning on principles to best integrate between these interventions over time.

It is worth noting, however, that multiple issues related to digital micro interventions still need to be resolved. First, research methodologies that can facilitate the evaluation of micro interventions are required. Micro randomized trials are one such methodology that could be of use here, especially when research questions are related to dynamic processes of change over time [66]. Second, methods for optimizing digital micro interventions and improving micro intervention suites need to be explored. Third, we need to understand the people and targets for which the delivery of digital micro intervention care is more or less suitable.

Much of the current innovation in micro interventions has made use of technological affordances as technology makes it more feasible to deliver digital micro interventions in various ways. However, again, it does not necessarily mean that this sort of intervention has to be digital by nature. Because technology is highly embedded in our lives, it is possible that digital capabilities such as automated monitoring, triggering, and instant messaging tools will be commonly integrated into care, making the differentiation between digital and nondigital tools less relevant.

Expanding the portfolio of behavioral and mental health interventions to include interventions that allow people to interact in various modalities, intensities, and styles is likely necessary to make such interventions available to the vast number of people who could stand to benefit from them. Digital micro interventions represent one example of such an expansion, as they are brief, focused, and potentially more appropriate for people who enter with various levels of motivation. We hope the model represented here will structure and speed the work in this area and improve the research and delivery of digital micro intervention care.

## Appendix

Multimedia Appendix 1 An example for an automated therapeutic narrative.
