# Supplementary material for: Digital Micro Interventions for Behavioral and Mental Health Gains: Core Components and Conceptualization of Digital Micro Intervention Care
Source: J Med Internet Res. 2020 Oct 29;22(10):e20631. doi: 10.2196/20631 (PMC7661243; doi:10.2196/20631)
Supplement: Multimedia Appendix 1 [file jmir_v22i10e20631_app1.pdf]

<u>**Appendix 1 – An example for an automated therapeutic narrative**</u>

In the following example, we present an automated therapeutic narrative that is based on a pre-written text. The narrative is presented to a parent who is enrolled to digital micro-intervention care that targets positive parenting practices. In this example, the therapeutic narrative and recommended intervention follows a parent report on child's misbehaviors after successful completions of two micro-interventions that are based on Table 3 levels of prioritization (first level: improving quality time through play; second level: social coaching). Content within "[]" represent values situated within a predetermined variable.

"Hi [parent_name], thank you for entering the program and reporting on [child_name]'s misbehaviors. We analyzed your data and notice your success in prior micro-interventions that focused on [incorporating positive interaction routines (e.g. quality time with); problem solving (e.g. helping your child doing homework)].

Your effort invested in prior steps helped you build a better relationship with your child, as part of your commitment to improve your parenting skills.

This now enables you to focus on directly coping with [child_name]'s noncompliance to your requests. Congratulations for keep making the conscious choice of investing effort in improving your parental skills! These skills are so crucial in helping [child_name] get by with his family, teachers, and friends. Here is a link to your new step: [URL]"
